# Molecules and Mechanisms Implicated in the Peculiar Antigenic Activation Process of Human Vγ9Vδ2 T Cells

**DOI:** 10.3389/fimmu.2014.00657

**Published:** 2015-01-05

**Authors:** Christelle Harly, Cassie-Marie Peigné, Emmanuel Scotet

**Affiliations:** ^1^Laboratory of Genome Integrity, Center for Cancer Research, National Cancer Institute, Bethesda, MD, USA; ^2^Department of Pathology and Laboratory Medicine, Perelman School of Medicine, University of Pennsylvania, Philadelphia, PA, USA; ^3^Unité Mixte de Recherche 892, Centre de Recherche en Cancérologie Nantes Angers, INSERM, Nantes, France; ^4^University of Nantes, Nantes, France; ^5^Unité Mixte de Recherche 6299, Centre National de la Recherche Scientifique, Nantes, France

**Keywords:** human γδ T lymphocytes, functions, antigenic activation, phosphoantigens, butyrophilin

## Abstract

In human beings, as well as in most non-human primates, the major peripheral γδ T cell subset, which accounts several percent of the whole lymphoid cells pool in adults, carries an heterodimeric TCR composed of Vγ9 and Vδ2 chains. Vγ9Vδ2 T cells are specifically and strongly activated by small organic pyrophosphate molecules termed phosphoantigens (phosphoAg). These low molecular weight compounds are metabolites that are produced by either microbes or endogenously, as intermediates of the mammalian mevalonate pathway, and can accumulate intracellularly during cell stress like transformation or infection. Despite the characterization of numerous natural and synthetic phosphoAg, the mechanism(s) underlying the unique and specific antigenic activation process induced by these compounds remains poorly understood. Activation is both TCR- and cell-to-cell contact-dependent, and results of previous studies have also strongly suggested a key contribution of membrane-associated molecules of primate origin expressed on target cells. The recent identification of B7*-related* butyrophilin (BTN) molecules CD277/BTN3A, and more precisely their BTN3A1 isoforms, as mandatory molecules in the phosphoAg-induced recognition of target cells by Vγ9Vδ2 T cells opens important opportunities for research and applications in this field. Here, we review the unusual and complex antigenic reactivity of human Vγ9Vδ2 T cells. We highlight the recent advances in our understanding of this process, and propose a model that integrates the type I glycoprotein BTN3A1 and its intracellular B30.2 domain as a physical intermediate implicated in the detection of dysregulated intracellular levels of phosphoAg and the sensing of cell stress by Vγ9Vδ2T cells. A better understanding of this mechanism will help optimize novel immunotherapeutical approaches that utilize the unique functional potential of this major γδ T cell subset.

## γδ T Cells as Mandatory T Cell Subsets or Evolutionary Relics?

T cells are critical component of the adaptive immune system and are essential for defense against foreign organisms and self-dysregulation. T cells can be divided into two lineages, according to the composition of their T cell receptor (TCR). While most αβ T cells recognize peptidic antigens (Ag) bound to highly polymorphic major histocompatibility complex (MHC) molecules and are qualified as conventional T cells, several T cell subsets react against conserved non-peptidic Ag. Like other adaptive immune effectors, these latter non-conventional or innate-like T cells express clonally distributed Ag recognition receptors, made of either αβ or γδ TCR subunits, which are associated to subunits of the CD3 signal transduction complex at the cell membrane. The ontogenic and developmental features of γδ T cells, which have been extensively reviewed elsewhere (1–4), will be summarized here. During thymic ontogeny, γδ T cells emerge before αβ T cells and are predominant at early stages of fetal development (embryonic days from 14 to 18 in mice). One of the striking hallmarks that distinguish γδ from αβ T cell subsets is their potential for TCR structural diversity, which is considerable despite a highly restricted V gene repertoire. However, this very limited combinatorial diversity of γδ TCR is efficiently counterbalanced by extensive junctional diversity, due to removal or addition of non-germline-encoded nucleotides at V-(D)-J junctions and alternate D segments reading frames, which allows the generation of a γδ TCR diversity greater than that of conventional αβ T cells (theoretically ~10^16^ in rodents and human). The extent of TCR junctional diversity can greatly vary from one γδ T cell subset to another, with several populations expressing highly conserved so-called “invariant” TCRs in some tissue locations, as it is the case for murine Vγ5Vδ1 or Vγ6Vδ1 T cell subsets. An important feature of γδ T cells is the preferential expression of TCR V regions in distinct tissue locations. In human beings, Vγ9Vδ2 T cells are preferentially found in the peripheral blood where they represent more than 80% of γδ T cell pool, and about 5% of the CD3^+^ cells in healthy adults. By contrast, Vδ2^−^ subsets, such as Vδ1^+^ and Vδ3^+^ subsets, are mainly detected in epithelial tissues (e.g., epidermis and mucosa), spleen, thymus, and liver. The preferential localization of γδ T cell subsets might be explained by their capacity to be activated and to expand upon recognition of specific ligands whose expression would be restricted to particular tissues, or to specific homing capacities acquired during intrathymic development (5). In agreement with a peripheral tissue expansion process, absolute numbers of human Vγ9Vδ2 T cells steadily increase in blood during the first years of life as the diversity of their TCR repertoire decreases.

Strikingly and in contrast to mice and humans, γδ T cells often represent the major circulating lymphocytes in cattle, sheep, pigs, and birds (6), which strengthen the idea that γδ T cells play a critical role in immune responses to cell stress and infections. However, since the initial characterization of γδ T cells in the late 1980s, the *raison d’être* of this mysteriously distinct subset of CD3^+^ T cells within evolved vertebrate species (such as primates and rodents), which already carry innate and adaptive immunity cell subsets, remains unclear. A recent study has revealed that the genetic programs for two primordial T cell-like lineages, oddly similar to αβ and γδ T cells, and one B cell-like lineage are found in several species of jawless vertebrates devoid of RAG recombinase and MHC molecules (7). It is not known whether a tripartite adaptive immune system was already present in a common vertebrate ancestor 500 millions years ago, and diverged since then along two distinct phylogenetic lineages, or it appeared two times independently by convergent evolution. Yet this finding strongly argues for a unique role of γδ T cells, as effectors of the transitional immunity endowed of unique functional properties and/or antigenic specificities.

## Functional Features and Antigenic Specificities of γδ T Cells

γδ T cells have been characterized for their ability to deliver a broad array of effector functions upon activation *in vitro* and *in vivo*. Numerous studies mostly performed in mice, human beings, and non-human primates, indicate that γδ T cells are implicated in the control of infectious (e.g., bacteria, virus, parasite) diseases, tumor development, homeostasis, wound healing, and tissue repair. The functional features of γδ T cells have been recently and extensively reviewed elsewhere (3, 4, 8) and will not be detailed in this review.

γδ T cells, including human Vγ9Vδ2 T cells, can directly kill and eliminate infected, activated, or transformed cells and contribute to pathogen clearance. In these physiopathological contexts, activated γδ T cells engage pathways associated to the release of cytotoxic and bacteriostatic molecules such as perforin, granzymes, granulysin, and defensins, death-inducing receptors and TNF-related apoptosis-inducing ligand receptors. γδ T cells are also able to produce modulatory cytokines that play a key role against intracellular pathogens and the promotion of inflammation (e.g., TNF-α, IFN-γ), extracellular bacteria, fungi (e.g., IL-17), and parasites (e.g., IL-4, IL-5, IL-13). γδ T cell-released cytokines and factors might have beneficial or deleterious effects according to the physiopathological context. As an example, activated γδ T cells are able to downmodulate immune responses (e.g., TGF-β, IL-10), to promote tissue healing, epithelia repair, and cell survival. Interestingly, studies have shown that activated γδ T cells can promote dendritic cells (DC) maturation through the release of type I and type II IFNs, which underlies their adjuvant role played for the control of infections (9–12). Unexpectedly, some γδ T cell subsets, like human Vγ9Vδ2 T cells, could also constitute a novel type of professional APC that would elicit CD8^+^/CD4^+^ αβ T cell responses through the acquisition of a DC*-like* phenotype upon antigenic activation (13). Whether or not such function is found in other human and murine γδ T cell subsets remains to be assessed. To date, none of the broad functional features described for γδ T cells is specific to this T cell subset. Conjugated attempts of many laboratories failed to clearly establish and define common functional features of γδ T cells that would basically distinguish them from conventional and innate-like αβ T cells. Taken together, these observations suggest that most of the key contribution of the functional responses displayed by activated γδ T cells might rather rely on the tight regulation of their kinetics of activation as well as the ability of these innate-like T cell subsets to be present “at the right time, in the right place.” The unique Ag specificities of γδ T cells could also significantly account for their “programed” distribution within organs and tissues and their striking evolutionary conservation aside from T and B cell subsets which also assemble their Ag-receptor genes through recombinatorial rearrangement.

One particularly attractive hypothesis to account for the remarkable species and inter-individual conservation of γδ T cells, as well as the lack of functional redundancy with αβ T and B cells, is that this former subset, like an intermediate “T–B hybrid” cell type, might be rather designed for an efficient and unique mode of recognition of a broad set of conserved native Ag (e.g., proteins, lipids, carbohydrates) or complexes. In such contexts, this set of Ag either directly interact with γδ TCR or are presented by non-polymorphic MHC*-related* or yet unknown presenting molecules. In line with this hypothesis, the structure of γδ TCR heterodimers suggests that these molecules display immunoglobulin (Ig)-like recognition features, which strengthen the idea for alternative modes of Ag recognition by γδ TCRs (14). This view is supported by both the diversity and the nature of γδ TCR agonist molecules already identified, as well as by the direct reactivity of γδ T cells and B cells against similar native molecules (e.g., F_0_-F_1_ ATP synthase, phycoerythrin) (15, 16).

γδ T cells are key players in the immune surveillance of cellular distress, owing to their general ability to recognize Self determinants that are frequently upregulated in contexts of inflammation, infection, or cancer. While γδ TCR contribute to detection of danger-associated molecular patterns, cognate interactions between γδ TCR and defined Ag have been reported in a few cases only. As for some other innate-like cell subsets, γδ T cells can be activated, in a cell-to-cell contact but classical MHC-independent manner, by small molecules or intact proteins without requirement for a processing similar to the conventional αβ T cells Ag. Strikingly, while γδ T cells can respond to different stimuli in various physiopathological contexts, a substantial fraction of the physiological murine and human γδ TCR ligands characterized so far fall into the large MHC class I-related molecules family (e.g., T10-T22, MICA/B, CD1c, CD1d, EPCR) (17–21). Moreover the activation of γδ T cells subsets by some of these molecules might be dependent on their ability to present specific ligands such as lipids (e.g., EPCR, CD1d).

## The History of Phosphoantigens and the First Identification of Natural Human Vγ9Vδ2 T Cell Agonist Molecules

**Figure 1:**
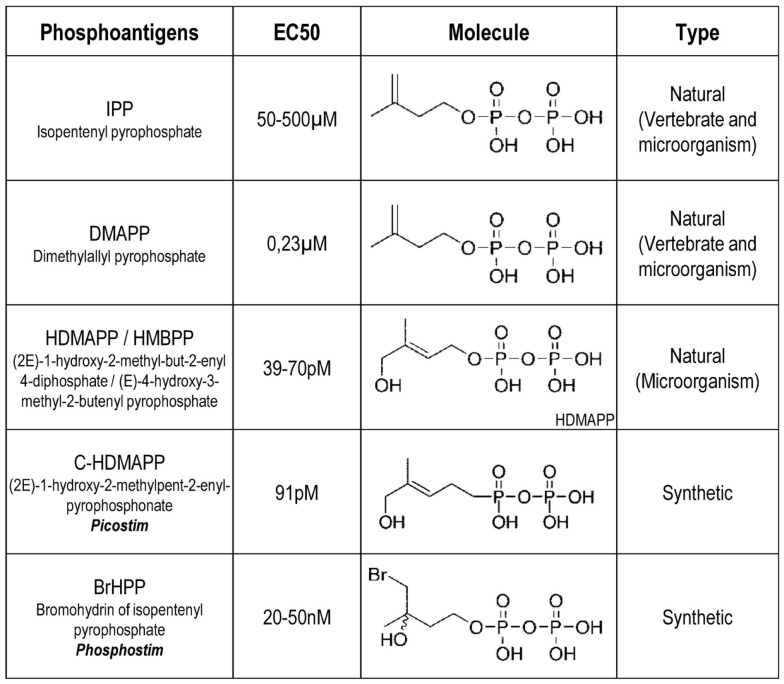
**Examples of characterized phosphoantigens that induce Vγ9Vδ2 T cell activation**. These listed phosphoAg are either from natural or synthetic origin. They induce different range of activation with EC_50_ value for Vγ9Vδ2 T cell activation that can vary between different human donors. From Ref. (64, 36, 39, 97).

In humans, as well as in most non-human primates, the major peripheral γδ T cell subset in adults carries a TCR composed of a Vδ2 chain systematically paired to a Vγ9 chain (using the nomenclature of Lefranc and Rabbitts (22), also referred to as Vγ2 by using the Seidman and colleagues nomenclature). This γδ T cell subset represents about 5% of CD3^+^ cells within human peripheral blood and more than 80% of the peripheral γδ T cell population in healthy adults. After the fortuitous discovery of γδ T cells and the subsequent generation of monoclonal antibodies (mAbs) specific for human γ or δ TCR chains, high frequencies of Vγ9Vδ2 T cells were reported within peripheral blood or lesions from patients infected by a variety of bacteria (e.g., *Mycobacterium leprae*, *Mycobacterium tuberculosis*) and protozoans. Accordingly, Vγ9Vδ2 T cells efficiently responded to mycobacterial extracts *in vitro*, which contained small non-peptidic compounds (protease-resistant) that also bind to lectins. The isolation and characterization of the stimulatory fractions within these extracts led to the identification of various agonist low molecular weight carbohydrate compounds that were structurally related to phosphoesters and depend on the presence of phosphate moieties for their bioactivity (23–26). IPP (isopentenyl pyrophosphate) from *M. smegmatis* was identified as the first natural agonist for Vγ9Vδ2 T cells. This compound, and its isomer DMAPP (dimethylallyl pyrophosphate), were called phosphoAg, a family of compounds including the *E coli* and *mycobacteria*-derived (2E)-1-hydroxy-2-methyl-but-2-enyl-4-diphosphate (HDMAPP), also known as (E)-4-hydroxy-3-methyl-2-butenyl pyrophosphate (HMBPP), and additional natural molecules produced by many microorganisms and plants (27–30) (Figure 1). Reports also indicated that Vγ9Vδ2 T cells are efficiently activated by a broad array of human tumor cells, such as lymphoma and carcinoma. The Vγ9Vδ2 T cells antigenic molecules from tumor cells were identified as IPP and DMAPP phosphorylated metabolites of the isoprenoid mevalonate (MVA) biosynthesis pathway, which is implicated in cholesterol synthesis (31). Accordingly, pharmacological MVA pathway inhibitors acting upstream (e.g., statins) or downstream (e.g., alkylamines; aminobisphosphonates, NBP) of phosphoAg biosynthesis, respectively, decrease or increase Vγ9Vδ2 T cell activation (32). Whereas most eukaryotic cells, fungi and archeabacteria produce isoprenoids through the MVA pathway, most of eubacteria, cyanobacteria, and algae protozoa synthesize isoprenoids through a related biosynthesis pathway referred to as the 1-deoxy-d-xylulose-5-phosphate (DOXP) or 2-C-methyl-d-erythritol 4-phosphate (MEP) pathway (33). HDMAPP produced through the DOXP pathway remains the most potent natural Vγ9Vδ2 activator so far identified (bioactivity of 0.1 nM, which is 30,000 times more potent than IPP). This suggests that this highly potent microbial phosphoAg, rather than endogenous phosphoAg such as IPP, would account for the highly sensitive sensing by Vγ9Vδ2 T cells of infected cells or pathogens using the DOXP/MEP pathway. The efficiency of the microorganisms recognition process by γδ T cells was linked to their DOXP/MEP isoprenoid synthesis pathway (34). Accordingly, the genetic manipulation of the DOXP/MEP pathway in bacteria, similarly to the pharmacologic manipulation of the MVA pathway in human cell lines, regulates the production of these microbial phosphoAg which resulted in the modulation of the antigenic activation of Vγ9Vδ2 T cells (34, 35).

Research groups have next attempted to define the chemical basis and structure-to-functions relationships for the antigenicity of phosphoAg. These studies first reported that the substitution of phosphate for the pyrophosphate moiety or the elimination of the double bond reduced antigenic bioactivity of these compounds (24). As synthetic phosphoAg have been further produced and tested (e.g., BrHPP for bromohydrin pyrophosphate), these various molecules were compared to the natural ones (Figure 1) and next classified from an antagonist or weak, medium to a strong agonist bioactivity on Vγ9Vδ2 T cell activation (27, 36). These studies indicate that agonist or antagonist bioactivity of these compounds correlates with the extent and kinetics of pyrophosphate dephosphorylation (also referred to as β-phosphate cleavage) and alteration of their organic segment. Interestingly, a fast extracellular acidification measured after cell exposure to soluble agonist phosphoAg was reported (37, 38). As the phosphonate moieties are less susceptible to chemical or enzymatic hydrolysis than their corresponding phosphate counterparts, some groups have also synthesized phosphonate and pyrophosphonate analogs that could have improved pharmacological properties linked to an increased stability in biological media (39).

The specific and efficient activation of primate Vγ9Vδ2 T cells induced by isoprenoid phosphorylated compounds provides an attractive unified explanation for the unique and broad reactivity of this T cell subset in infectious and tumor contexts. However, the mechanism(s) of this highly conserved and uncommon specific antigenic recognition process by Vγ9Vδ2 T cells has remained puzzling and ill defined.

## A Peculiar Mode of Vγ9Vδ2 T Cell Activation Induced by Phosphoantigens?

The unique ability of primate Vγ9Vδ2 T cells to specifically sense very subtle variations of phosphoAg levels in various physiopathological contexts such as infections and cancer highlighted the importance of the role played by the Vγ9Vδ2 TCR molecules in this process. Indeed, correlative studies have initially reported the specific activation of human T lymphocytes expressing a Vγ9Vδ2 TCR by Daudi Burkitt’s lymphoma cells and mycobacteria (40, 41) and a direct contribution of germline residues specific to Vγ9, Vδ2, and JγP elements to this recognition process (42). Altogether, these studies suggested that the antigenic activation of Vγ9Vδ2 T cells induced by phosphoAg is mediated by germline-encoded segments of the γδ TCR. The γδ TCR dependence of phosphoAg-induced T cell reactivity, also supported by the effects of γδ TCR blocking mAbs (23, 24, 26), has been finally demonstrated by the results of Vγ9Vδ2 TCR transfer experiments (43). Indeed, the transfection of a Vγ9Vδ2 TCR in human Jurkat T cells enabled these cells to produce IL-2 in response to Daudi human tumor cells, mycobacterial extract, and soluble phosphoAg. Taken together with additional observations (44–46), this indicated that expression of Vγ9Vδ2 TCR is necessary and sufficient for the efficient sensing of identical, or structurally related, Ag from mycobacterial extracts, tumor cells, NBP-sensitized cells and synthetic soluble phosphoAg. Accordingly, events and signaling pathways classically triggered in T lymphocytes upon TCR-dependent activation (e.g., Erk and p38 MAPK pathways) were also reported for phosphoAg-induced activation of human Vγ9Vδ2 T cells (47–50).

Various mechanisms have been proposed to account for the Vγ9Vδ2 TCR-dependent sensing of phosphoAg that triggers strong activation of γδ T lymphocytes. In a first hypothesis, soluble phosphoAg, as fully conserved native molecules, which are released by microbes or mammalian target cells (e.g., from tumor or NBP-treated cells) could directly, and specifically, interact with Vγ9Vδ2 TCR heterodimers to trigger γδ T lymphocyte activation. This process would rely on both the bioactivity and the concentration of the released compounds. This direct interaction model is supported by: (i) the 3D structural features of a Vγ9Vδ2 TCR together with the results of γδ TCR transfer/mutagenesis experiments that revealed the existence of a putative “binding” groove that could accommodate small (1–3 kDa) negatively charged phosphoAg (14) and, (ii) a mandatory role of the junctional region of the TCR γ chain (51) and the contribution of key conserved residues such as: lysine residue in the γ chain CDR3 loop, an arginine residue in CDR2δ, and an aliphatic amino acid residue in CDR3δ (52, 53). However, all attempts to biophysically or biochemically demonstrate any direct phosphoAg/Vγ9Vδ2 TCR interactions have failed so far (14, 54) and additional key observations, such as the cell-to-cell contact requirement for the activation of Vγ9Vδ2 T cells by soluble phosphoAg (55, 56) do not favor a direct recognition process.

## The Hunt for Unknown Phosphoantigen-Presenting Molecule(s)

On the basis of these last observations, non-exclusive molecular events were proposed to account for the necessity of cell-to-cell contacts for phosphoAg-induced Vγ9Vδ2 T cell activation, such as: (i) a “stabilization” of native or modified membrane-bound phosphoAg on target cells surface that would be required for protection against degradation by extracellular phosphatases, (ii) a topological clustering/aggregation of phosphoAg and/or their co-localization with key membrane-associated molecules (e.g., adhesion, NKR) for an efficient cross-link of the γδ TCR.

The critical contribution of species-specific Ag-presenting cells has been evidenced, meaning that only cell-to-cell contact with human, and some non-human primate cells, are able to trigger a phosphoAg reactivity of human Vγ9Vδ2 T cells (57, 58). This restriction indirectly suggested the requirement for species-specific determinants and, as primate phosphoAg-sensitized cell lines lose their ability to efficiently activate Vγ9Vδ2 T cells upon protease treatments, these key surface determinants were characterized as membrane-associated proteins (59). Several adhesion and costimulation molecules (e.g., ICAM-1, CD166) have been proposed to account, at least partially, for these requirements (57, 58). However, human Vγ9Vδ2 TCR transfer into murine T cell lines was shown to be sufficient to trigger phosphoAg reactivity against human, but not rodent target cells (60, 61) (C. Harly & E. Scotet, *unpublished obervations*). Additionally, macaque Vγ9Vδ2 TCR tetramers were shown to bind to human, but not mouse cell surface upon phosphoAg treatment. In agreement with the contribution and the protein nature of key cell surface determinants, this binding was abrogated by protease pre-treatments of the target cells (59). Altogether, these results underlined the critical requirements of primate cell membrane-expressed determinants of protein nature, specifically recognized by Vγ9Vδ2 T cells. The contribution of these unknown molecule(s) was further supported by a study uncovering the role of all the CDRs of Vγ9Vδ2 TCR in the recognition of phosphoAg-sensitized cells, thus suggesting a large contact surface with a putative antigenic complexes/molecules, and not only small phosphorylated compounds (62). Additionally, the existence of “phosphoAg-presenting molecules” was supported by the characterization of photoaffinity prenyl pyrophosphate Ag (analogs of HMBPP), designed to form covalent bounds with close proximity molecules after UV-treatment. This study suggests that such molecules stably associate to broadly distributed, functionally non-polymorphic, and not known Ag-presenting molecule(s) on human target cells and activate Vγ9Vδ2 T cells (63). At last, the generation and the functional characterization of various synthetic compounds analogous to phosphoAg has revealed that some of them were not only unable to efficiently activate Vγ9Vδ2 T cells, but could specifically interfere with the stimulating activity of phosphoAg (37, 63, 64). Similarly, IPP could efficiently inhibit the stable association of photoaffinity prenyl pyrophosphate Ag to primate target cells (63). These observations could reflect the existence of competition mechanisms established between phosphoAg (e.g., IPP) and analogs for a limited number of binding sites on the same unidentified molecule(s) expressed by target cells.

PhosphoAg recognition has been described to be an extremely rapid process (~10 s) and is not abrogated by glutaraldehyde fixation of the target cells (36, 55). In contrast, the antigenic activation of Vγ9Vδ2 T cells by NBPs or alkylamines is indirect and mediated by the intracellular accumulation of IPP (31, 65, 66). It remains unclear how intracellular IPP is detected at the cell surface. Whether phosphoAg are transported, exported (e.g., intracellular IPP) outside or internalized inside (e.g., extracellular HMBPP) the target cells, specifically or not, anchored or adsorbed on target cell surface, whether they interact with yet unknown ubiquitous surface molecule or whether they rapidly regulate the expression/conformational changes of determinants that are detected by Vγ9Vδ2 TCR remains open.

Taken together, the previous findings would tend to rule out a “simple” direct recognition process of phosphoAg by Vγ9Vδ2 T cells, and lead to hypothesize the existence of a “phosphoAg-presenting molecule(s)” of protein nature, ubiquitously expressed on the surface of primate cells. However, such molecule(s) was never identified, and, as we will see in the second part of this review, more recent works strongly suggest an alternative model for phosphoAg sensing by human Vγ9Vδ2 T cells.

## BTN3A1, an Ubiquitous Cell Surface-Expressed Human Butyrophilin Molecule, Plays a Mandatory Role for the Activation of Human Vγ9Vδ2 T Cells Induced by Phosphoantigens

Recently, our group has clearly demonstrated for the first time the specific and mandatory role played by BTN3A1, a type I glycoprotein expressed on target cells, in the phosphoAg-induced reactivity of human Vγ9Vδ2 T cells (60). BTN3A1, -A2, and -A3 constitute the BTN3A (also known as CD277) subfamily of butyrophilins (BTN) molecules. These three isoforms are encoded by three distinct genes, found in human and some non-human primates. *Btn* genes constitute a subgroup of at least 10 genes in most species. Eleven of them have been identified in mouse, and 13 in human, where they are located in the MHC class I region of chromosome 6p. Notably, BTN family is member of the Ig superfamily and shares structural homology with B7 family members at extracellular domain level (mostly composed of Ig-like domains referred to as IgV and IgC domains) (67). Phylogenetically, BTN molecules share a common ancestor with the B7 family, initially suggesting that they could have immunological functions (68).

The functions of BTN molecules as well as their molecular partners remain ill defined. The eponymous BTN gene, *BTN1A1*, is highly expressed in the lactating mammary glands (secretory epithelium) and represents the major protein associated with fat droplets in milk. BTN1A1 has been reported to mainly play a role in the regulation of the amount of lipids and size of droplets expressed in milk of mammals (69). This function is linked to its intracellular B30.2 domain, which has been shown to bind xanthine oxydoreductase and stabilize its association with the milk fat globule membrane (70). A few studies have reported immunological functions for some human BTN members such as BTN1A1, BTN2A2 and BTN3A molecules that can regulate cellular immunity and T/NK cells activation (67, 71–75). As compared to BNT1A1, human BTN2A and BTN3A are widely expressed in many tissues (76). The ectodomains of the three isoforms of BTN3A consist of two domains, so-called IgV and IgC that have a very high homology (>95%). On the other hand, the intracellular domain B30.2 (PRY/SPRY) is only present in BTN3A1 and BTN3A3 and poorly conserved. BTN3A1 is ubiquitously expressed in human beings (75, 77) and homologs are found in all primates carrying Vγ9Vδ2 T cells (78). Strikingly, BTN3A orthologs are not found in the rodent lineage, which also lacks Vγ9Vδ2 T cell counterparts specific for phosphoAg. The emergence of TCR Vγ9, Vδ2, and BTN3 genes with eutherian placental mammals has been recently reported (79). This recent study suggests a strong evolutionary functional link between the expression of Vγ9Vδ2 TCR and BTN3 proteins.

Our study shows a mandatory role for the BTN3A1 isoform in the specific detection of human distressed cells (e.g., tumor and mycobacteria infected cells) by human Vγ9Vδ2 T cells, which strongly suggested that BTN3A1 molecule represent a major species-specific determinant regulating the antigenic reactivity of human Vγ9Vδ2 T cells (60). This work demonstrates that BTN3A1 does not act as a key costimulatory or adhesion molecule but as a mandatory protein for the Vγ9Vδ2 TCR-dependent phosphoAg-mediated recognition of target cells. Furthermore, our data indicate that BTN3A1 expression is necessary for the recognition of human target cells by baboon Vγ9Vδ2 T cells, and that antigenic activation of baboon Vγ9Vδ2 T cells, induced by either human or baboon cells is abrogated by a blocking anti-BTN3 mAb (C. Harly & E. Scotet, *unpublished observations*), which is fully in line with the evolutionary functional link between the expression of Vγ9Vδ2 TCR and BTN3 proteins. It will be interesting to extend these observations to other Vγ9Vδ2 expressing species, including the non-primate ones, such as alpacan (79) to either strengthen or challenge this functional link. Importantly, several groups (61) (C. Harly & E. Scotet, *unpublished obervations*) have reported that the expression of BTN3A1 is probably not sufficient to induce the recognition of rodent target cells by human Vγ9Vδ2 T cells, even when co-expressed with key human adhesion molecules (e.g., ICAM-1). In contrast, transferring a 27.4 megabases region of the human chromosome 6p including BTN3A1 could confer to a mouse cell line the ability to activate Vγ9Vδ2 T cells in the presence of phosphoAg (61, 80). This suggests that additional species-specific partner molecules encoded on the human chromosome 6p, together with BTN3A1, are required for ensuring its functional activity.

## The Intracellular B30.2 Domain of BTN3A1 Binds Phosphoantigens and Mediates Their Sensing by Vγ9Vδ2 T Cells

While other molecules involved in the phosphoAg-mediated recognition of target cells by Vγ9Vδ2 T lymphocytes remain to be identified, the physical and chemical features of BTN3A1 already shed light on possible mechanism(s) taking part to this process. BTN molecules comprise two extracellular Ig-like domains, a single pass transmembrane domain, and, for some members including BTN3A1 and BTN3A3, a B30.2 (SPRY/PRY)-related intracellular domain (67). The mandatory role played by BNT3A1 isoform during antigenic activation of Vγ9Vδ2 T cells, but not BTN3A2 or BTN3A3, first suggested that the highly conserved extracellular region (>95%) of BTN3A1 unlikely accounts for its functional specificity in phosphoAg-mediated activation. In line with this assumption, we next showed, by swapping the domains between BTN3A isoforms, that the intracellular portion of BTN3A1 is necessary for BTN3A1 to mediate phosphoAg stimulation of Vγ9Vδ2 T cells, and is sufficient to confer this properties to the inactive isoform BTN3A3 (60). Therefore this finding highlights a crucial and specific contribution for the intracellular B30.2 domain of BTN3A1.

These results have been very recently confirmed and extended by a joined study from the group of E. J. Adams and ours. In this work, a set of structural, molecular and cellular approaches has been performed to unambiguously demonstrate the direct interaction between phosphoAg and the N-terminal part of the intracellular domain B30.2 of BTN3A1, through a positively charged pocket (81). Each of the pyrophosphate compound that activate Vγ9Vδ2 T cells bind the B30.2 domain of BTN3A1, but not the B30.2 domain of BTN3A3, and their agonist potency is directly correlated with their binding intensity. Strikingly, comparative sequence analysis between the B30.2 domains of BTN3A1 and BTN3A3 isoforms identified a putative Histidine residue specific to BTN3A1 (replaced by an Arginine at this position in BTN3A3). Structural, biochemical, and functional assays show that this residue is required for the binding of phosphoAg to BTN3A1 B30.2, and their Vγ9Vδ2-stimulating activity. These observations, which have been confirmed by other groups (64, 82), indicate that internal sensing of changes in phosphoAg metabolite concentrations by BTN3A1 molecules represent a critical step in Vγ9Vδ2 T cell detection of infection and tumorigenesis.

## Phosphoantigens Act Intracellularly and Therefore are Not *Bona Fide* Vγ9Vδ2 T Cell Antigens

**Figure 2:**
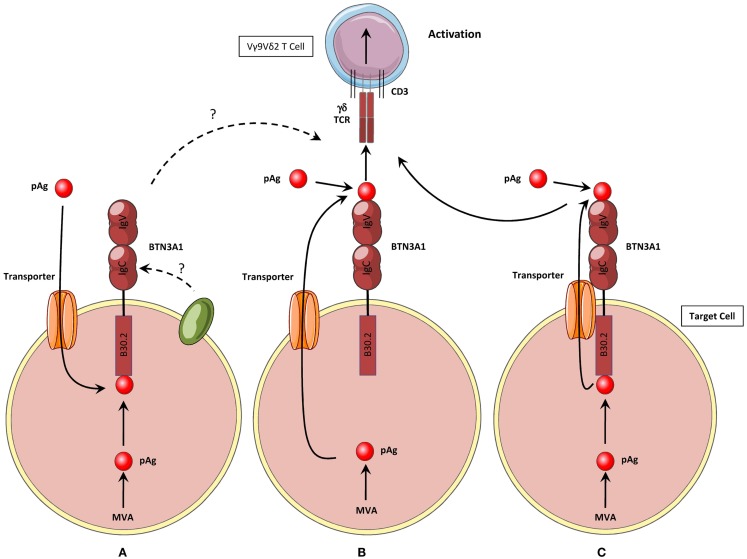
**Three hypothetical activation mechanisms for the induction of human Vγ9Vδ2 T cells activation by phosphoantigens**. **(A)** Hypothesis #1: phosphoAg are produced intracellularly or internalized by unidentified membrane transporter(s). They interact with the intracellular B30.2 domain of BTN3A1, which induces non-exclusive key modifications such as conformational changes of the protein, modifications of its membrane topology, recruitment/exclusion of molecular partners. These modifications are then sensed by Vγ9Vδ2 T cells, leading to their functional activation. **(B)** Hypothesis #2: Intracellular phosphoAg are exported from the cell by unidentified membrane transporter(s). Both extra- and intracellular phosphoAg interact with the extracellular part of CD277/BTN3A. phosphoAg are presented by the extracellular IgV domain, as antigenic complexes to the Vγ9Vδ2 TCR, which leads to γδ T cell functional activation. **(C)** Hypothesis #3: this model shares mechanisms from both hypothesis #1 and #2 models. Intracellular phosphoAg interact with the intracellular B30.2 domain of BTN3A1. They are then exported from the cell by BTN3A1 itself, or a membrane transporter(s) associated with BTN3A1. Both extra- and intracellular phosphoAg can then interact with the extracellular IgV domain of BTN3A1 and trigger Vγ9Vδ2 T cell activation.

The direct physical and functional link established between the intracellular phosphoAg and the transmembrane protein BTN3A1 represents a tremendous advance in deciphering how primate Vγ9Vδ2 T cells can exquisitely sense the dysregulation of intracellular phosphoAg levels by scanning the surface of distressed target cells. However, many key issues remain to be solved to fully understand the fine modalities of this process, summarized in Figure 2A. How is the intracellular interaction between pyrophosphate compounds and intracellular B30.2 domain translated to the cell surface? Which cell surface determinants are finally detected by Vγ9Vδ2 T cells? How exogenous phosphoAg efficiently “sensitize” target cells through BTN3A1, while the negative charge of their phosphorylated moities unlikely allows them to passively cross the plasma membrane, and their mode of action was proposed to be independent of any active processing machinery (55, 63)?

A recent study from the group of G. De Libero has proposed an attractive model for BTN3A1 function, which would resolve some of these issues. Endogenous and exogenous phosphoAg would be directly first loaded on the extracellular portion of BTN3A1 and this complex would next directly interact with the Vγ9Vδ2 TCR (80) (Figure 2B). This study provide molecular and cellular evidences for a direct interaction between phosphoAg, the extracellular IgV domain of BTN3A1, and the Vγ9Vδ2 TCR therefore supporting the appealing assignment of BTN3A1 as a classic Ag-presenting molecule for pyrophosphate compounds. However, this model appears to be insufficient to explain several key experimental observations, such as the specific requirement for BTN3A1 isoform, but not for the BTN3A2 and BTN3A3, whereas the extracellular domain of the three proteins is highly homologous, especially within the area composed of the candidate binding residues for phosphoAg, which are shared between BTN3A1 and BTN3A2. More importantly, this model also does not take in account the mandatory role of the intracellular B30.2 domain of BTN3A1 in this process. A hypothesis that could reconcile some of the features seemingly contradictory between these two models would be that BTN3A1 itself is a transporter molecule, or closely associated with one. In this model, BTN3A1 would interact with both intracellular and extracellular phosphoAg through, respectively, its B30.2 domain and its extracellular IgV domain. This latter one would next serve for the presentation of both type of phosphoAg to Vγ9Vδ2 T cells (Figure 2C). However, the ability of the extracellular part of BTN3A1 to bind phosphoAg and interact with the Vγ9Vδ2 TCR has not been confirmed by either biochemical or functional analysis (61, 64, 81, 82) (C. Harly & E. Scotet, *unpublished obervations*). Taken together, the studies performed so far indicate that phosphoAg-induced sensing of target cells is mediated by the BTN3A1 intracellular B30.2 domain for both endogenous and exogenous phosphoAg (Figure 2A). As these results failed to demonstrate any direct contribution (e.g., interactions) of the extracellular region of BTN3A1 in this process, they would rather support a model in which phoshoAgs are not directly recognized by Vγ9Vδ2 T cells, and therefore are not *bona fide* Vγ9Vδ2 Ag.

An important issue raised from this assumption concerns the requirements for the internalization of exogenous phosphoAg within the cells, through the plasma membrane. Early evidence did not support the existence of such a process: (i) the negative charge of phosphoAg does not allow them to passively cross the plasma membrane, (ii) phosphoAg do not seem to require any active internalization or processing for triggering Vγ9Vδ2 T cell activation (55, 63), (iii) the very fast kinetics of Vγ9Vδ2 T cell activation induced by phosphoAg suggest they are readily active at the extracellular level (37, 38, 47, 49). The mode of intracellular action of negatively charged exogenous phosphoAg has been recently addressed in a cellular approach (64). In this elegant study, synthetic pro-phosphoAg was designed to allow their passive diffusion through the plasma membrane, in an inactive protected state. After cleavage of the protective groups by intracellular esterases, active phosphoAg were released and were unable to leave the cytoplasm. The results clearly show that these highly bioactive compounds bind the intracellular domain of BTN3A1. Additionally, the intracellular uptake of pro-phosphoAg was confirmed by a long-term effect of the pro-drug on target cell sensitization after washes. Therefore, these results strongly support an unified mode of action of phosphoAg to trigger Vγ9Vδ2 T cell activation, regardless of their endogenous, exogenous, intracellular, and extracellular origin.

Thus, phosphoAg would act intracellularly in target cells and their direct interaction with the B30.2 domain of BTN3A1 would be next sensed by Vγ9Vδ2 T cells. This model, as well as the alternative ones, relies on the existence and the contribution of additional molecular player(s), involved in either the uptake of extracellular phosphoAg, or the release of intracellular phosphoAg (Figure 2). Whether this process is active or not, phosphoAg-specific or not, and can impact the quality and the magnitude of the functional activation of Vγ9Vδ2 T cells remains to be determined. The identification of yet unknown membrane-associated transporter molecule(s) should greatly help understanding of the mode of phosphoAg action.

## Re-Organization of Cell Surface Molecules Induced by Interactions between Phosphoantigens and the BNT3A1 Intracellular B30.2 Domain?

This model of intracellular detection of phosphoAg raises the key question of the nature of molecular events implicated in the sensing of this process by Vγ9Vδ2 T cells. This binding of phosphoAg to the B30.2 domain of BTN3A1 should be somehow “translated” to the cell surface of target cells, in order to be detected by Vγ9Vδ2 T cells. An interesting clue for deciphering this mechanism comes from the observation that some mAbs specific of the extracellular region of BTN3A can precisely mimic the phosphoAg-induced recognition of target cells by Vγ9Vδ2 T cells (60, 83). Additionally, some anti-BTN3 mAbs abrogate both phosphoAg- and agonist anti-BTN3 mAbs-induced recognition of target cells by Vγ9Vδ2 T cells. This suggests similar mechanisms of sensitization of target cells induced by both phosphoAg and agonist anti-BTN3 mAbs. Interestingly, the sensitization of target cells induced by agonist anti-BTN3 mAbs requires neither the presence of phosphoAg nor the expression of intracellular B30.2 domain. Accordingly, each BTN3 isoform, comprising or not a B30.2 domain, was equally able to trigger the functional activation of Vγ9Vδ2 T cells (60, 82). The structural analysis of extracellular region of BTN3A1 with agonist ScFv mAb revealed a ~20 Å displacement as measured between V domains, induced upon binding of the agonist mAb (78). Altogether these observations strongly suggest that agonist anti-BTN3 mAbs can probably induce some key BTN3A structural modifications, similar to the effects triggered by phosphoAg. However, agonist anti-BTN3 mAbs in complex with the extracellular part of BTN3, are neither sufficient to activate Vγ9Vδ2 T cells when plastic-coated or expressed on the cell surface of rodent cells, nor able to interact with Vγ9Vδ2 TCR in solution (60, 78, 81). Though still conflicting (61, 80), these results suggest that the direct recognition of either anti-BTN3 mAbs in complex with extracellular BTN3 or conformational changes/crosslinking of the extracellular domain of BTN3 induced after binding of these agonist anti-BTN3 mAbs might not be sufficient to fully account for the antigenic activation of Vγ9Vδ2 T cells.

Thus, the topological remodeling of some cell surface key determinants, involving the aggregation or the exclusion of specific membrane proteins likely represents an important step for this stress-sensing process by Vγ9Vδ2 T cells. This is supported by recent FRAP (*Fluorescence Recovery After Photobleaching*) experiments showing that anti-BTN3 mAbs significantly decreases the mobility of BTN3A isoforms on the cell surface, regardless of the composition of their intracellular domain (60, 81). Importantly, similar effects were observed on human target cells with NBP-induced intracellular accumulations of phosphoAg, in correlation with the expression of a functional B30.2 domain. Hence, these results provide a strong link between the reduced surface mobility of BTN3A on human target cells and their detection by Vγ9Vδ2 T cells. The mechanism(s) implicated in such membrane diffusion alterations and the physiological relevance of these events still need to be experimentally further addressed. It would be interesting to determine whether human BTN3A molecules, when expressed on the cells surface of rodent cells, are also immobilized after phosphoAg or anti-BTN3 mAbs treatments, despite the inability of such cells to trigger functional activation of human Vγ9Vδ2 T cells. Such experiments should help further understand the link between BTN3A1 reduced mobility induced by phosphoAg and Vγ9Vδ2 T cell activation, as well as determine whether some key primate-specific molecules are required for: (i) translating the phosphoAg/B30.2 interactions into this reduced BTN3A mobility process, (ii) BTN3A immobilization itself, or, (iii) Vγ9Vδ2 T cell activation induced following sensing of this alteration of BTN3A mobility.

**Figure 3:**
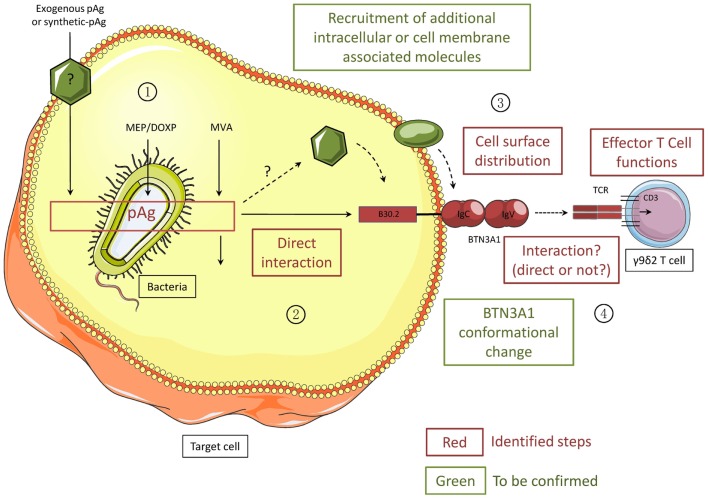
**Different steps of the activation process of human Vγ9Vδ2 T cells induced by phosphoantigens**. All the steps/molecules colored in red have been identified while the green ones will need to be confirmed. PhosphoAg (pAgs) accumulate intracellularly as metabolites of the endogenous mevalonate pathway (MVA) or the microorganism MEP/DOXP pathway. Exogenous pAg can also be internalized through yet undefined membrane transporter(s). PhosphoAg directly interact with the N-terminal portion of the intracellular B30.2 domain of CD277/BNT3A1. Intracellular partner molecules could be recruited either by BTN3A1 or by phosphoAg. Following PhosphoAg/B30.2 interaction, the conformation of BTN3A1 is altered and the cell surface distribution of BTN3A1 together with yet unknown other molecular partners is modified. These exquisite signals are sensed by Vγ9Vδ2 T cells, in a TCR-dependent manner, leading to the functional activation of γδ T cells. Whether the Vγ9Vδ2 TCR directly interacts with BTN3A1 remains unclear.

These FRAP experiments present essential evidence that the simple interaction(s) of small intracellular metabolites, like phosphoAg, with the ubiquitously expressed intracellular BTN3A1 B30.2 domain can substantially affect the global topological organization of this cell surface-expressed type I glycoproteins. The observation that BTN3A1 B30.2 crystals dissolve upon phosphoAg soaking (81) raises the possibility that the interaction(s) between phosphoAg and this B30.2 domain could result in conformational rearrangements leading to this membrane mobility alterations. This hypothesis is supported by nuclear magnetic resonance spectroscopy experiments revealing some major chemical shift perturbations upon phosphoAg binding in the B30.2 domain, not only within the B30.2 domain, but also in the upstream membrane proximal region (64). Therefore, these recent results open the possibility that such induced conformational changes could be translated from the intracellular domain of BTN3A1 to its extracellular domain, similarly to the “inside-out” signaling process already observed with integrins (84), and result in a global cell surface remodeling (Figure 3).

## Which are “Real” TCR Ligands for Human Vγ9Vδ2 T Cells?

While a lot of attention has recently been brought onto the mechanism(s) linking phosphoAg and BTN3A1, the molecule(s) that could finally interact with the Vγ9Vδ2 TCR and trigger T cell activation remain(s) unidentified. It is particularly unclear whether BTN3A1 is directly recognized, alone or in molecular complexes, or if this molecule indirectly plays a key role for the recognition of Vγ9Vδ2 TCR ligand(s) at the target cell surface. Several candidate molecules have been previously proposed as Vγ9Vδ2 T cells Ag accounting for the specific recognition of tumor or infected cells, in various pathological contexts. These candidate molecules were from bacterial [e.g., GroEL HSPs (85)], SEA (86), viral [e.g., HSV-1 glycoprotein I (87), or Self origin [e.g., *F*_0_–*F*_1_ ATP synthase (15)], ULBP4 (88), MSH2 (89), HSP60 (90)]. Mitochondrial *F*_0_–*F*_1_ ATP synthase was a particularly interesting candidate as the ectopic cell surface expression of this mitochondrial complex was reported on tumor cells. *F*_0_–*F*_1_ ATP synthase also binds a delipidated form of apolipoprotein A-I, which is required for optimal Vγ9Vδ2 T cell activation. Further studies showed the direct recognition of these molecules by the Vγ9Vδ2 TCR (15), a process that can be modulated by MHC class I molecules (91). Because *F*_0_–*F*_1_ ATP synthase was shown to interact with some phosphoAg, this molecule was proposed as a presenting molecule for phosphoAg (92) but this property has not been yet confirmed. Interestingly, the recent characterization of Vγ9Vδ2 T cell subsets amplified *in vitro* following contact with *M. BCG*-infected DCs has shown that only a subset of phosphoAg-responsive Vγ9Vδ2 T cells were actually responsive to *M. BCG* (93). TCR repertoire analysis of this T cell subset revealed a restricted diversity in Vδ2 CDR3 sequences, as compared to IPP-reactive Vγ9Vδ2 T cells. This study suggested that the reactivity of these Vγ9Vδ2 T cell subsets toward *M. BCG* could be mediated by non-phosphoAg molecules. Similar observations were made in pulmonary tuberculosis patients (94). The specificity of these subsets was next characterized which led to the identification of new protein/peptides of mycobacterial origin (e.g., OXYS, DXS2, Rv2272) (94, 95) that can interact with Vγ9Vδ2 TCRs and activate a large fraction of Vγ9Vδ2 T cells isolated from peripheral blood of tuberculosis infected patients but not γδ T cells of healthy donors.

Therefore, these recent studies provide attractive evidences that some Vγ9Vδ2 TCRs might not be designed for the unique detection of phosphoAg/BTN3A1-linked stress signals. However, antagonist anti-BTN3 mAbs have been shown to abrogate the recognition of *M. BCG*-infected cells (60). Even if this result does not directly address the esssential requirement for BTN3A1 expression, and its intracellular B30.2 domain, in the reactivity of Vγ9Vδ2 T cells against *M. BCG*, it strongly underlines an important function for this BTN in this process. As the “polyreactivity” of various Vγ9Vδ2 T cell clones toward various antigenic molecules has not been determined so far, it is therefore difficult to exclude or include them in a general model of Vγ9Vδ2 T cell activation by phosphoAg. An interesting hypothesis is that each of these Vγ9Vδ2 TCR ligands expressed on the surface of stressed target cells is not individually detected by Vγ9Vδ2 T cells, but become activator as part of a complex involving several Vγ9Vδ2 TCR ligands, as suggested by the results from the study reporting the recognition of the *F*_0_–*F*_1_ ATP synthase/ApoA1 complex (15). In agreement with this idea, BTN3A1 could regulate the formation or the topology of Vγ9Vδ2 antigenic complexes on the cell surface.

## Physiological Implications for the Detection of Both Modified-Self and Non-Self by Vγ9Vδ2 T Cells

Vγ9Vδ2 T cells have the ability to discriminate between self and modified-self, by detecting subtle changes of phosphoAg levels, due to increased MVA pathway activity and/or uptake of exogenous phosphoAg, within stressed, transformed, or infected target cells. How Vγ9Vδ2 T cells can make this subtle distinction is not well understood, but a fine control of such capacities is essential to avoid any deleterious effect linked to the strong effector functions of Vγ9Vδ2 T cells (e.g., cytolysis) in case of an inappropriate activation. Importantly, as also suggested for other T cell subsets, the recognition of altered determinants, from either self or non-self origin, by the TCR complex does not fully reflect the “true” reactivity of these T lymphocytes and the ability of these ligands/TCR interactions to trigger functional responses. Indeed, the TCR-mediated reactivity of Vγ9Vδ2 T cells against target cells not solely depends on the expression levels of Ag but also relies on the fine tuning of their activation threshold by costimulatory/adhesion molecules and activating/inhibitory NK receptors (47, 57, 58, 96).

The interaction of phosphoAg with the intracellular B30.2 domain of the ubiquitous BTN3A1 molecule, at a 1:1 molar ratio (64, 81), which would be subsequently translated to the cell surface, offers a simple option for the quantitative sensing of endogenous phosphoAg within target cells by Vγ9Vδ2 T cells. However, the MVA pathway, which produces the endogenous phosphoAg metabolites (e.g., IPP), is constitutively engaged at different levels of activity in healthy cells, according to their physiological functions. For example, highly proliferating cells such as activated proliferating T cells display some metabolic characteristics that are very similar to those of stress-altered cells, including an upregulated MVA pathway. As several additional molecular players remain to be identified to fully decipher the mechanisms of phosphoAg sensing by Vγ9Vδ2 T cells (Figure 3), the complexity of the currently proposed model already offers many levels of regulation, such as the expression levels of molecules regulating phosphoAg/B30.2 interactions and/or BTN3A1 conformational/topological changes, as well as the availability of BTN3A1 or Vγ9Vδ2 TCR ligands themselves. Therefore, following their identification, it will be critical to study the expression of each of these molecules in both physiological and pathological contexts. As an hypothesis, BTN3A isoforms that are devoid of any phosphoAg-binding B30.2 domain could exert inhibitory functions by competing with BTN3A1 for the interaction with molecular partners (e.g., ectopic *F*_0_–*F*_1_ ATP synthase, ApoA1 or other TCR ligands). To first test this, the expression levels of the different isoforms of BTN3A will need to be further measured and compared, not only in different healthy cell subsets (75) but also in various altered contexts.

Besides their ability to quantitatively sense variations of endogenous phosphoAg levels in target cells, Vγ9Vδ2 T cells also efficiently discriminate between weak agonist phosphoAg that are produced by the endogenous MVA pathway (e.g., IPP) and strong agonist ones which are produced by the microorganism DOXP/MEP pathway (e.g., HMBPP). Isothermal titration calorimetry has shown that the potency of phosphoAg correlates with their binding affinity for the intracellular B30.2 domain of BTN3A1 (64, 81). According to biochemical and biophysical studies, the binding of phosphoAg on the B30.2 domain would rely on their mandatory phosphate moiety, which interacts with a unique binding site in the B30.2 domain, and could be modulated by the nature of their organic solvent (64, 81). It remains unclear how such binding affinities are in turn quantitatively translated to the target cell surface and next detected by Vγ9Vδ2 T cells. One possibility is that the stability of these interactions could directly impact on the stability of the induced BTN3A1 conformational/topological changes. Another hypothesis is that phosphoAg direcly or indirectly recruit partner molecules, according to their biochemical properties. As the chemical reactivity of phosphoAg has been reported as a key requirement for Vγ9Vδ2 T cells activation and the kinetics of the dephosphorylation step correlates with their bioactivity, the importance of the contribution of this β-phosphate cleavage step will also need to be further analyzed. Interestingly, some phosphoAg analogs that are resistant to β-phosphate cleavage are not only unable to activate Vγ9Vδ2 T cells but also abrogate their activation induced by phosphoAg (37). These observations suggest that phosphoAg and their analogs compete for binding/interacting sites (e.g., B30.2 domain) and that β-phosphate cleavage is required for an additional step still to be identified.

## Concluding Remarks

Primate Vγ9Vδ2 T cells are endowed with unique reactivity patterns against a broad range of stressed cell targets, including infected and tumor cells. This property has been mainly attributed to a specific recognition of dysregulated phosphoAg in various pathological contexts, as well as an apparently diversified set of proteins and peptides of Self and non-Self origins that could directly interact with the Vγ9Vδ2 TCR. Despite the lack of obvious common feature between all these antigenic determinants, several Vγ9Vδ2 TCR ligands of Self origin have been linked to the cell metabolism, and their expression or localization could be regulated by transformation or infection. Because metabolic dysregulation represents a general feature of various cell distress events, including transformation, infection and injury, the activation of Vγ9Vδ2 T cells induced by this type of antigenic determinants would represent a unique and efficient strategy allowing the early detection of many pathological contexts. It will be crucial in future studies to understand by which mechanisms such molecules of diverse nature and origin can trigger and regulate Vγ9Vδ2 T cell antigenic activation, and whether all these molecules are recognized individually, or as part of a general mechanism of detection of distressed-self and non-self, involving phosphoAg, BTN3A1, and various other molecules yet to be identified. Our attempts to integrate the results of previous experimental data on the modalities of Vγ9Vδ2 T cell activation induced by phosphoAg, highlighted by the recent identification of BTN3A1 as mandatory player in this process, led to an incomplete, yet already complex model of phosphoAg sensing by Vγ9Vδ2 T cells. Against all expectations, phosphoAg sensing by Vγ9Vδ2 T cells is an indirect process (Figure 3), involving several intermediate players that remain to be identified.

## Conflict of Interest Statement

The authors declare that the research was conducted in the absence of any commercial or financial relationships that could be construed as a potential conflict of interest.
